# An acute viral hepatitis epidemic: does ultrasound help the pediatrician?

**DOI:** 10.1186/s13104-021-05510-1

**Published:** 2021-03-10

**Authors:** Sadaf Arooj, Muhammad Umer Mukhtar, Farnaz Abbas

**Affiliations:** 1 King Edward Medical University, Lahore, Pakistan; 2 Department of Pediatrics, King Edward Medical University, Lahore, Pakistan

**Keywords:** Acute viral hepatitis, Ultrasonography, Gallbladder wall thickening, Pericholecystic edema

## Abstract

**Objective:**

Acute viral hepatitis (AVH) caused by hepatitis virus A and hepatitis virus E is one of the many epidemics that plague third world countries like Pakistan. The serological tests required for the diagnosis of acute viral hepatitis may be unavailable or unaffordable to the denizens of a developing country like Pakistan. In such a scenario, the clinical manifestations and the ultrasonographic findings are the only diagnostic criteria usually present and these can be used to support a clinical diagnosis. This study aims to judge the utility of ultrasound in the diagnosis of AVH.

**Results:**

Among the forty-seven subjects of this study, gall bladder wall thickening (GWT) was the most common radiological finding seen in 39 (82.9%) patients. Pericholecystic edema was the second most common finding, seen in 31 (65.9%) patients. Starry sky appearance of the liver was observed in 30 (63.8) patients. Hepatomegaly and ascites were seen in 28 (59.5%) and 25 (53.2%) of the patients, respectively. The ultrasonographic findings of GWT, pericholecystic edema, and starry sky appearance of the liver were the most common ultrasonographic findings associated with AVH.

**Supplementary Information:**

The online version contains supplementary material available at 10.1186/s13104-021-05510-1.

## Introduction

Hepatitis means inflammation of the liver which may result from infectious causes like microorganisms or from non-infectious agents like drugs, alcohols, or hepatotoxic substances. Acute viral hepatitis (AVH) is most commonly caused by infection by hepatitis A or E virus and ranges from subclinical disease or self-limited symptomatic disease to fulminant hepatic failure [1]. The term AVH subsequently in this article shall refer to the acute symptomatic disease caused by hepatitis A virus (HAV) or hepatitis E virus (HEV).

Viral hepatitis is a leading killer worldwide. WHO estimated that infections with hepatitis viruses result in more number of deaths than that are caused by notorious epidemics like HIV, tuberculosis, or malaria [2]. Viral hepatitis after being long neglected has gained yet more visibility, with the global viral hepatitis death count increasing from 0.89 million to 1.45 million, between the years of 1990 and 2013 and viral hepatitis crowning itself as the seventh leading cause of death worldwide [3]. There have been only a few community-based epidemiological studies on AVH in Pakistan. There are many risk factors predisposing the Pakistani population to viral hepatitis epidemics such as low socioeconomic status, poor sanitation and water supply, poor hygienic conditions, low vaccination trends, population congestion and poor health care delivery system. These regretful conditions are further exploited upon by the viruses during the Monsoon season which causes seasonal rains and floods from June to September resulting in waterborne epidemics of AVH, with children presenting a particular challenge [4, 5].

The symptomology of AVH in pediatric patients includes fever, jaundice, vomiting, dark-colored urine, pale stools, abdominal pain, nausea, anorexia and malaise. Associated physical findings include hepatomegaly, splenomegaly, ascites and edema. Laboratory markers of liver function like ALT, AST are elevated along with serum bilirubin [6]. There is strong correlation between the ultrasound findings and the clinical severity of the disease. Even the lab values of ALT, AST and total bilirubin show association, not only with the clinical severity of the disease but with the radiological picture as well [7].

A provisional diagnosis of hepatitis may be made on the classical clinical presentation and liver markers and confirmation can be made on the basis of serological analysis [7]. But the cost of serological investigations is high and they are not easily available in developing countries. Thus, the noninvasive diagnostic technique of ultrasonography can be used to support diagnosis made on clinical features. In addition, reliable data on epidemiology, clinical and radiological profile for AVH is deficient. Thus, this study aims not only to highlight the importance of radiological features in the diagnosis of hepatitis but also to provide proper information needed for planning schemes and strategies for the prevention and cure of AVH.

## Main text

### Methods

#### Setting and time of study

This study was conducted from June to September, 2019 in the Department of Radiology, King Edward Medical University, Pakistan.

#### Study design

Cross-sectional study.

#### Inclusion criteria

All male and female patients of the pediatric population (neonate to 13 years old) presenting with acute onset of symptoms of fever, abdominal pain, nausea, deranged LFTs and a serological diagnosis of acute hepatitis caused by either HAV or HEV were included as subjects.

#### Exclusion criteria

Patients with age more than 13 years, chronic hepatitis B or C were excluded (only patients with acute symptoms and serological diagnosis of HAV or HEV were included. HAV and HEV do not progress to chronicity and thus chronic hepatitis was effectively excluded). Additionally, patients with liver masses, liver failure, acute cholecystitis, and cirrhosis or any other disease that is endemic in Pakistan were excluded.

#### Data collection procedure

Informed written consent from parents of patients was taken. Ultrasound Abdomen and Pelvis was performed. Machine Toshiba I style model 2012 was used. Clinical history was taken and biodata was noted.

#### Imaging and laboratory parameters

Hepatomegaly is a pathologic increase in the size of the liver. On ultrasonography, the presence of hepatomegaly in a pediatric patient is judged on the basis of knowledge about the size of the liver in a healthy child of the same age. Gallbladder wall thickening (GWT) is defined as an increase in the thickness of gallbladder wall greater than 3 mm. Pericholecystic edema is fluid around the gallbladder on ultrasound. Starry sky appearance is defined as the presence of bright periportal triads on ultrasonography. Normal reference range is < 1.2 mg/dL for total bilirubin and ≤ 40 IU/L for ALT and AST [8].

#### Data analysis procedure

Data was entered and analyzed using IBM Statistical Packages for Social Sciences 24. Quantitative variables like age were presented as mean ± standard deviation. Qualitative variables like gender were presented as frequency and percentage.

### Results

Out of the 47 patients that met our inclusion criteria, 30 were male and 17 were female patients with male to female ratio of 1.7:1. Our youngest patient was 1 year old and oldest was 14 years old.

Patients had varying constellations of clinical symptoms (Table 1). The most common presentations were jaundice and passing dark colored urine.

**Table 1:** Clinical presentation of the patients

Clinical symptom	Present	Absent
Fever	37 (78.7%)	10 (21.3%)
Anorexia	33 (70.2%)	14 (29.8%)
Nausea and vomiting	31 (66%)	16 (34%)
Abdominal pain	36 (76.6%)	11 (23.4%)
Jaundice	43 (91.5%)	4 (8.5%)
Dark urine	43 (91.5%)	4 (8.5%)
Light stools	26 (55.3%)	21 (44.7%)
Right hypochondriac pain	34 (72.3%)	13 (27.7%)

**Table 2:** Ultrasonographic findings of the patients

Ultrasonographic findings	Present	Absent
Hepatomegaly	28 (59.5%)	19 (40.5%)
Liver texture (Starry Sky Appearance)	30 (63.8%)	17 (36.2%)
Pericholecystic edema	31 (65.9%)	16 (34.1%)
Gallbladder wall thickening	39 (82.9%)	8 (17.1%)
Gallbladder sludge	5 (10.6%)	42 (89.4%)
Ascites	25 (53.2%)	22 (46.8%)
Pleural effusion	6 (12.8%)	41 (87.2%)

GWT was found to be the most common ultrasound finding of AVH, present in 39 (82.9%) patients (Table 2). Pericholecystic edema was the second most common finding, seen in 31 (65.9%) patients. Starry sky appearance the third most common radiological finding. These findings were followed by hepatomegaly and ascites in terms of frequency. Pleural effusion and gall bladder sludge were seen in a few patients only (Tables 2, 3).

**Table 3:** Lab values

Laboratory parameters	Values
Total bilirubin (mg/dL)	5.3 ± 3.2 (0.40–13.70)
ALT (IU/dL)	565.7 ± 585.1 (35–2347)
AST (IU/dL)	647.3 ± 608.4 (55–2595)
WBC	7.853 × 103ML ± 3.2120

Table 3 shows various lab parameters that were recorded. All lab parameters showed derangement due to AVH.

### Discussion

The incidence of viral hepatitis is rising globally as well in Pakistan. Seasonal rains and floods that occur during the Monsoon season take advantage of poor sanitary conditions in third world countries like Pakistan and result in epidemics of fecal-orally transmitted diseases such as AVH [5].

Definite diagnosis of AVH requires serological analysis for antibodies but these serological tests are expensive and practically out of reach for the plenteous poor of the developing world. On the other hand, the technique of ultrasonography is easily available and hence clinical diagnosis of hepatitis made upon classical features like jaundice, pale stools, right hypochondriac pain may be supplemented by ultrasound findings. To this purpose, many studies have evaluated and emphasized the importance of ultrasonographic findings of AVH [7].

A case–control study done in a tertiary care hospital of India by Maurya et al. included patients of all ages. They found out that hepatomegaly (86.6%) and GWT (75.8%) were the most significant ultrasound findings in AVH [7].

GWT is one of the most important features of AVH. There are multiple causes of GWT that are suggested in the literature. This sonographic feature may be non-specific alone but is highly sensitive for the diagnosis of acute hepatitis [9, 10]. The exact pathophysiology of GWT in acute hepatitis can be explained by three possible mechanisms. First, there is hepatocyte injury leading to decreased bile production. This results in decreased gallbladder volume and a relative increase in gallbladder wall thickness. Secondly, there may be direct inflammation of layers of the gallbladder wall causing GWT. The third explanation is that hepatocyte necrosis causes inflammation to be spread to the gallbladder resulting in GWT [11].

In our study, GWT was present in 82.9% of the patients and pericholecystic edema in 65.9% of the patients. In a study conducted by Sudhamshu, 84% of the patients showed GWT. In a similar study by Sudhamshu et al., GWT was seen in 91% of the patients. Both these studies show a frequency of GWT almost identical to ours. A recent study of AVH patients by Maurya et al. conducted in 2019, showed GWT in 75.8% of the cases, further stressing this finding [7, 11, 12].

The prognostic value of gallbladder wall thickness was specifically assessed by Ahn et al. This study showed direct relation of gallbladder wall thickness and liver enzyme elevation. They divided the patients into two groups, Group A included patients having increased gallbladder wall thickness and Group B with normal gallbladder wall thickness. Patients were studied in terms of ultrasonographic features, liver enzymes, hospital stay duration, and follow up ultrasound. They concluded that liver enzyme elevation and duration of hospital stay were significantly associated with increased gallbladder wall thickness [13].

Another important imaging parameter in AVH is bright periportal triads giving a ‘starry sky appearance’. In our study, it was seen in 63.8% of the patients. This typical appearance of the periportal triads is due to edema of liver cells that decreases the echogenicity of the liver. The edema further causes an alteration in sound properties and accentuates the walls of portal venous channels [14].

In a study done by Shin et al., 71–75% of the patients revealed starry sky appearance whereas GWT was found in 77–100% of the patients of severe acute hepatitis. They concluded that both starry sky appearance and gallbladder wall thickness is important in predicting severe acute hepatitis A [15].

Hepatomegaly is also an important finding in ultrasonography of hepatitis patients. It was present in 28 (59.5%) of our patients. Girish et al. and Modi et al. found hepatomegaly in 76% and 30% of their patients respectively [6, 16].

Overall, ultrasound has high sensitivity for AVH (as shown by the high rate of occurrence of ultrasonographic findings in our study and previous studies) but the specificity is low as many other conditions may also come up with these ultrasonographic findings of GWT, starry sky appearance of the liver, etc. [17, 18]. We advise the clinician (especially the one working in underprivileged circumstances) to compensate for this low specificity of ultrasound for AVH by using his clinical acumen to correctly judge the symptomatology of his patient; whether it is indicative of AVH or not. AVH has a very distinct symptomatology. The clinical picture along with the highly sensitive findings of GWT, pericholecystic edema and starry sky appearance of the liver, will allow the clinician to arrive at a correct diagnosis of AVH in case of unavailability of serologic tests.

Our study revealed a predictably low pattern of vaccination in the Pakistani population (Additional file 1: Table S1). Only 72.3% of the patient has received vaccination. This shows that lack of vaccination trend is another cause of the occurrence of epidemics of AVH year after year in Pakistan, despite claims of mass vaccination campaigns by the government.

The differential diagnosis of AVH are acute cholecystitis and acute pancreatitis which may show up with ultrasonographic finings similar to that of AVH. However, both of these diseases show other important USG findings which help us to differentiate them from AVH. The most sensitive ultrasound finding in acute cholecystitis is the presence of cholelithiasis in combination with the sonographic Murphy sign. GWT (> 3 mm) and pericholecystic fluid are secondary findings [19]. Ultrasound findings in acute pancreatitis are swollen hypoechoic parenchyma of pancreas with pancreatic necrosis and peripancreatic collections [20]. Hepatitis B and C do not have the ultrasound findings that are associated with HAV or HEV and instead may show normal ultrasound appearance of the liver to increased or heterogenous hepatic echogenicity [21].

### Conclusion

There is a significant correlation between acute infection of the liver by the hepatitis A and E viruses and the development of ultrasonographic findings. Consistent evidence from this and the prior studies is sufficient to conclude that ultrasonographic findings especially gallbladder thickening, starry sky appearance, and pericholecystic edema are corroborating findings for acute viral hepatitis caused by HAV and HEV and can be used to supplement clinical diagnosis in the case of unavailability of serological tests (Additional file 2).

## Limitations

Our study had a few limitations. The first being its small sample size. Secondly, we couldn’t follow the patients after recovery otherwise prognostic factors could have been estimated as well. Thirdly, our study had no control group. Thus, this study reflects observations expressed in the form of percentages. A better study for the future should incorporate a larger sample size with follow up imaging, and should employ ROC curve methodology to illuminate the correlation between ultrasonographic findings and AVH more strongly.

## Supplementary Information

**Additional file 1:Table S1**. Vaccination Trend.

**Additional file 2: Table S2.** Data set 1. This data set contains the different variables on which the conclusions of this study were based.

## Data Availability

The datasets used and/or analysed during the current study are available from the corresponding author on reasonable request.

## Abbreviations

AVH: Acute viral hepatitis
GWT: Gallbladder wall thickening
HAV: Hepatitis A virus
HEV: Hepatis E virus
