# Supplementary material for: An acute viral hepatitis epidemic: does ultrasound help the pediatrician?
Source: BMC Res Notes. 2021 Mar 10;14:95. doi: 10.1186/s13104-021-05510-1 (PMC7944630; doi:10.1186/s13104-021-05510-1)
Supplement: Supplementary file 1 — Additional file 1:Table S1. Vaccination Trend. [file 13104_2021_5510_MOESM1_ESM.docx]

The following table illustrates the vaccination trends and socioeconomic trends in the subjects of this study.

**Vaccination and Socioeconomic trends**

|  | **Yes** |
| --- | --- |
| Vaccination Status | 34(72.3%) |
| History of Contact | 15(31.9%) |
